# An analysis of cerebral amyloid angiopathy based on samples from human brain bank

**DOI:** 10.3389/fimmu.2026.1933760

**Published:** 2026-08-18

**Authors:** Yizhou Zhang, Mengyao Ye, Shixiong Mi, Yi Zhou, Qihan Yu, Jiayi Liang, Shiyi Wang, Qian Yang, Yuhong Su, Huixian Cui, Xiaowei Ma, Juan Du

**Affiliations:** 1 Department of Human Anatomy, Human Brain Bank, Hebei Key Laboratory of Neurodegenerative Disease Mechanism, Hebei Medical University, Shijiazhuang, China; 2 Department of Neurology, The First Hospital of Hebei Medical University, Shijiazhuang, China

**Keywords:** Alzheimer’s disease neuropathologic change, cerebral amyloid angiopathy, human brain bank, microglia, TREM1

## Abstract

**Background:**

Cerebral amyloid angiopathy (CAA) is a common age-associated cerebrovascular disease marked by amyloid-β (Aβ) accumulation in cerebral vessel walls, and it is tightly linked to Alzheimer’s disease (AD) and cognitive impairment. However, the specific mechanism in CAA progression remains poorly elucidated.

**Methods:**

We analyzed 49 autopsy samples obtained from the Human Brain Bank of Hebei Medical University, collected basic pathological data and explored the correlations between CAA and Alzheimer’s disease neuropathologic change (ADNC). Transcriptomic sequencing was performed on 21 occipital lobe specimens to screen differentially expressed genes underlying CAA and its pathological subtypes.

**Results:**

Neuropathological examination identified CAA pathology in 29 of 49 community donors (59.18%). Both CAA type and stage were significantly correlated with ADNC. In particular, CAA type 1 was closely associated with AD pathology and cognitive decline. Transcriptome analysis revealed that the GO functional enrichment was mainly concentrated in immune response-activating and -regulating signaling pathways. Triggering receptor expressed on myeloid cells 1 (*TREM1*) gene was involved in the amplification of immune signals, and it was expressed more strongly in the CAA1 subtype.

**Conclusion:**

CAA is not simply an independent cerebrovascular disorder but also serves as a critical synergistic risk factor for ADNC progression. *TREM1* is a potential gene that could be associated with a more severe pathological progression of CAA1 type.

## 1 Background

Cerebral amyloid angiopathy (CAA) is a common sporadic cerebral small vessel disease characterized by progressive β-amyloid (Aβ) deposition within the leptomeningeal and cortical vasculature, including small arteries, arterioles and capillaries (1). Vascular Aβ accumulation induces vascular fibrosis, endothelial dysfunction, luminal stenosis and increased vessel fragility, thereby triggering cerebral microbleeds, superficial siderosis, white matter hyperintensities and life-threatening intracerebral hemorrhage (2). CAA can be frequently observed in cognitively normal elderly, but also frequently in patients with Alzheimer’s disease (AD) (3). AD represents the most prevalent age-related neurodegenerative disorder (4), characterized neuropathologically by extracellular Aβ-enriched senile plaques and intracellular neurofibrillary tangles (NFTs) composed of hyperphosphorylated tau protein. CAA and AD neuropathological changes (ADNC) share core pathogenic mechanisms centered on disrupted Aβ metabolism, strongly supporting the existence of interactive pathological cascades between these two amyloid-driven disorders. Some researches reported that the prevalence of CAA varies among different countries and regions. The prevalence of neuropathologically confirmed CAA case range is very wide (eg., 0% - 79% in normal elderly) (5–11). Therefore, clarifying the epidemiological and pathological profiles of CAA in community-based Chinese population is critical to complement CAA heterogeneity and refine population-specific disease characteristics.

The pathological interplay between CAA and ADNC is further reinforced by shared genetic susceptibility, predominantly mediated by the *apolipoprotein E* (APOE) genotype. The *APOE* ϵ4 allele facilitates cerebral amyloid aggregation by impairing interstitial Aβ clearance, increasing vulnerability to both AD and CAA, whereas the *APOE* ϵ2 allele specifically elevates hemorrhagic risk in CAA patients (12). Despite these similarities, Aβ exhibits distinct deposition patterns: parenchymal plaque accumulation predominates in the hippocampus and association cortices in AD, while CAA is defined by exclusive vascular Aβ deposition (13). Aβ deposition within capillaries distinguishes two CAA subtypes: CAA type 1 presents capillary Aβ involvement, while type 2 lacks capillary deposition. CAA type 1 induces capillary occusion and cerebral perfusion disturbances, which likely contribute to cognitive decline in AD patients. Notably, perivascular Aβ deposits surrounding capillaries may extend into the parenchyma, a hallmark termed “dyshoric changes” that reflects blood-brain barrier (BBB) dysfunction (14). The compromised BBB not only impairs peripheral inflammatory mediators to infiltrate the brain parenchyma, exacerbating tau hyperphosphorylation, neuroinflammation and neuronal loss, and ultimately accelerating ADNC and cognitive dysfunction (15). Therefore, the mechanisms by which vascular amyloidopathy accelerates ADNC via disrupting BBB function remain to be further elucidated.

Accumulating evidence indicates that the immune system plays an active and sophisticated role in the initiation and progression of CAA (16, 17). On the one hand, resident immune cells alleviate amyloid pathology by eliminating Aβ deposits through phagocytosis and enzymatic degradation, thereby stabilizing cerebrovascular homeostasis and limiting disease progression. On the other hand, aberrant overactivation of neuroimmune responses induces persistent perivascular inflammation, which disrupts BBB integrity and exacerbates microvascular damage.

As the primary resident innate immune cells of the central nervous system (CNS), microglia display distinct activation patterns during CAA pathogenesis, characterized by morphological transformation, upregulated phenotypic markers, and unique spatial distribution. This specialized cellular response enables microglia to distinguish vascular Aβ from parenchymal Aβ deposits, likely through distinct receptor recognition systems (18). Moderately activated microglia exert protective functions by secreting Aβ-degrading enzymes, including neprilysin and insulin-degrading enzyme, and promoting perivascular Aβ clearance via microglial migration along the perivascular space (19). However, overactivated microglia release pro-inflammatory cytokines such as IL-6 and IL-1β to induce endothelial dysfunction, elevate complement expression to facilitate vascular complement deposition, and trigger NOX-mediated oxidative stress to injure vascular smooth muscle cells (20). Sustained microglia-mediated neuroinflammation further compromises BBB stability, increases microvascular permeability, and impairs glymphatic drainage function. These pathological alterations block interstitial Aβ clearance, accelerate parenchymal Aβ accumulation and tau hyperphosphorylation, and promote neuronal apoptosis, which synergistically drive ADNC progression and cognitive deterioration. A self-amplifying pathogenic loop is consequently formed, in which vascular Aβ deposition induces microglial inflammatory activation, and chronic immune disturbance further aggravates amyloid retention and vascular degeneration. Therefore, targeted regulation of microglial cell activity may be a potential strategy for alleviating the immune-inflammatory response in CAA.

Accordingly, the present study conducted comprehensive neuropathological and transcriptomic analyses based on post-mortem brain specimens. We aimed to characterize the pathological spectrum and subtype distribution of CAA in a community-derived Chinese elderly cohort, quantify the correlation between CAA pathological severity and ADNC burden, and further explore immune-related molecular signatures underlying CAA pathogenesis and heterogeneity.

## 2 Materials and methods

### 2.1 Human brain samples

Human brain tissues and corresponding demographic data were acquired from the Brain Bank of Hebei Medical University, China. All 49 autopsy specimens were collected from 2019 to 2026. Demographic information included age, gender, post-mortem delay (PMD), Everyday Cognitive (Ecog) score. Collected brain samples were processed in accordance with the standard (21, 22). Neuropathological diagnosis was performed to characterize cerebral lesions, particularly for neurodegenerative diseases including AD, CAA, LATE, etc. Transcriptome sequencing was only performed on samples with frozen brain tissue and acceptable RNA quality (RNA Integrity Number, RIN≥6.0 measured by Agilent 2100 Bioanalyzer). Transcriptomic sequencing was conducted on occipital tissues from 21 samples, consisting of 8 control cases and 13 CAA cases (**Supplementary File 1**). Immunochemistry staining was performed on 9 samples, including 3 controls and 6 CAA samples. This study protocol was approved by the Institutional Ethics Committee of Hebei Medical University (Approval No. 2025008).

### 2.2 Staining

The donated brain tissues were fixed in 10% neutral buffered formalin for four weeks. For neuropathological assessment, tissue blocks were dissected from each brain, followed by dehydration and paraffin embedding. Paraffin sections were cut at a thickness of 7 μm and stained with hematoxylin-eosin (HE), immunohistochemistry or special histochemical staining. In brief, for immunohistochemistry, the sections were dewaxed in xylene and rehydrated via a graded ethanol series, followed by antigen retrieval and incubated with endogenous peroxidase blocking solution at room temperature for 10 min. Primary antibodies diluted in PBS containing 5% goat serum were applied and incubated overnight at 4°C. The second day, horseradish peroxidase-conjugated goat anti-rabbit/mouse secondary antibodies were added and incubated at room temperature for 1 h. Signals were visualized with DAB chromogen, followed by hematoxylin counterstaining, dehydration through graded ethanol, and mounting with mounting medium. Primary antibodies included Aβ (DAKO, 1:200), phosphorylated tau (Thermo, 1:200), ɑ-synuclein (Novocastra, 1:200), phophorylated TDP43 (Affinity, 1:2000), p62 (BD, 1:200). Special stainings procedures comprised Congo red staining and Gallyas silver staining.

### 2.3 Neuropathological analysis of CAA, ADNC and comorbidities

CAA was subtyped into categories following Thal’s guidelines (23). The deposition of Aβ within cortical capillary was referred to CAA type 1. CAA type 2 referred to cases with leptomeningeal and cortical arteries Aβ deposition without capillary Aβ related. CAA progression was further classified into three topographical stages. Stage 1 was confined to leptomeningeal and neocortical parenchymal vessels. In Stage 2, amyloid pathology spread to allocortical regions and the cerebellum. Stage 3 was marked by additional involvement of the thalamus, basal ganglia, pons and medulla oblongata. The diagnosis of ADNC was evaluated based on the National Institute on Aging and Alzheimer’s Association (NIA-AA) standard system proposed by Montine T.J (24)., which involves assessing the pathological changes and their severity of senile plaques (A score), neurofibrillary tangles (B score), and neuritic plaques (C score). Senile plaques were classified into five stages based on their distribution and development patterns, and then converted into three scores (A1, A2, and A3) according to the severity. Neurofibrillary tangles were divided into six grades and converted into three scores (B1, B2, and B3) based on the severity. Neuritic plaques were classified into three grades based on the density (C1, C2, and C3). The combination of ABC score corresponded to different categories of ADNC, namely, None (N), Low (L), Intermediate (I), and High (H). Among them, none or low ADNC indicate that the sample was unlikely to have AD, while intermediate and high ADNC suggested a high probability of AD in the sample. The pathological diagnostic criteria for other related neurodegenerative diseases were adopted from published references (25–29).

### 2.4 Cognitive level assessment

Cognitive performance was evaluated with the Ecog scale (30). This validated informant-based scale assessed everyday cognitive decline covering seven domains: memory, language, semantic knowledge, visuospatial abilities, planning, organization, and divided attention. Each of the 39 items was scored on a four-point scale (1=no change, 2=occasionally worse, 3=consistently a little worse, 4=consistently much worse). The average total score ranged from 1 to 4, and higher scores indicated more severe cognitive impairment. Evaluations were completed by close family members or long-term caregivers. The Ecog scores were divided into three levels: normal (≤1), mild cognitive impairment (1-2), or dementia (>2) (31, 32).

### 2.5 *APOE* genotyping analysis

Genotyping for *APOE* alleles (ϵ2, ϵ3, ϵ4) was performed by genotyping two single-nucleotide polymorphisms, rs7412 and rs429358. Genomic DNA was extracted from blood specimens, and the detection was completed by RuiBiotech Co., Ltd.

### 2.6 Transcriptome analysis of CAA and CAA subtypes

Different genes were analyzed between control and CAA groups, and among control, CAA2 and CAA1 groups. The threshold value of |logFC| > 0.585 and *p* value < 0.05 was set as the differentially expressed genes. Gene Ontology (GO) and Kyoto Encyclopedia of Genes and Genomes (KEGG) enrichment analyses were performed to annotate the biological functions of differentially expressed genes.

### 2.7 Immunochemistry staining of different gene in CAA subtypes (TREM1)

TREM1 protein expression was detected on occipital lobe slices derived from three control cases, three CAA type 2 cases and three CAA type 1 cases. Immunohistochemical staining was performed following the protocol described in section 2.2, with TREM1 primary antibody (Boster, 1:50) applied for labeling.

### 2.8 Statistical analyses

Independent samples t-test was employed for the comparison of two groups of continuous variables that met the assumptions of normality and homogeneity of variance. The Mann-Whitney *U* test was used for comparisons between two groups when the continuous variables did not follow a normal distribution. Welch’s t-test was employed for the comparison of continuous variables that followed a normal distribution but exhibited unequal variances. One-way analysis of variance (ANOVA) was used to compare continuous variables among multiple groups when the assumptions of normality and homogeneity of variance were met. For data that deviated from a normal distribution, the Kruskal-Wallis *H* test was employed. In cases where the data were normally distributed but violated the assumption of equal variance, Welch’s ANOVA was performed. Spearman correlation analysis were used for evaluate CAA pathological changes, ADNC conditions, demographic data and transcriptome correlation analysis. Age was used as a control factor in the analysis of CAA type, CAA stage, combination of ABC score, A score, B score, C score and Ecog score. All statistical analyses were performed using SPSS 26.0. *p*<0.05 was regarded as statistically significant. Transcriptome data processing and analysis were performed using R software (Version 4.3.3) and Weishengxin platform (https://www.bioinformatics.com.cn, an online platform for data analysis and visualization).

## 3 Results

### 3.1 The demographic data and pathological characteristics of total samples

Basic clinical and pathological characteristics of the 49 samples are summarized in **Table 1**. The mean age of total samples was 75.24 ± 15.20 years; there were 33 men (67.35%) and 16 women (32.65%), respectively; the mean time of PMD was 9.42 ± 7.21 h; 8 samples (17.02%) were *APOE ϵ4* allele carriers and 7 samples (14.29%) were *APOE ϵ2* allele carriers. Common comorbidities included AD (30.61%), LATE (57.14%), arteriolosclerosis (73.47%), microinfarction (32.65%) and microhemorrhage (38.78%) et al. Regarding Ecog scores in the total samples, 33 (67.35%), 4 (8.16%) and 12 (24.49%) samples had a score ≤1, 1-2 and >2, respectively (**Table 1**).

**Table 1:** Clinical and pathologic characteristics of samples classified by CAA status.

Characteristics	Amount of participants (% of total)			*p* Value
	Total samples	No CAA	CAA	
	n = 49	n=20	n=29	
Age, mean [SD], y	75.24 [15.20]	66.30 [17.93]	81.41 [9.01]	< 0.001
Gender				
Male	33 (67.35)	13 (65.00)	20 (68.97)	0.771
Female	16 (32.65)	7 (35.00)	9 (31.03)
PMD				
Time, mean [SD], h	9.42 [7.21]	9.66 [8.15]	9.26 [6.62]	0.632
< 12 h	39 (79.59)	17 (85.00)	22 (75.86)	0.726
12-24 h	8 (16.33)	2 (10.00)	6 (20.69)
> 24 h	2 (4.08)	1 (5.00)	1 (3.45)
APOE				
* APOE ϵ4* allele*	8 (17.02)	0 (0.00)	8 (28.57)	0.015
* APOE ϵ2* allele*	7 (14.29)	3 (15)	4 (13.79)	1.000
Comorbidity				
AD	15 (30.61)	1 (5)	14 (48.28)	0.001
LATE	28 (57.14)	4 (20.00)	24 (82.76)	< 0.001
ARTAG	24 (48.98)	7 (35.00)	17 (58.62)	0.104
LBD	8 (16.33)	4 (20.00)	4 (13.79)	0.700
FTLD	2 (4.08%)	1 (5.00)	1 (3.45)	1.000
AS	36 (73.47)	9 (45.00)	27 (93.10)	0.001
Infarction	15 (30.61)	3 (15.00)	12 (41.38)	0.064
Microinfarction	16 (32.65)	3 (15.00)	13 (44.83)	0.035
Large hemorrhage	3 (6.12)	1 (5.00)	2 (6.90)	1.000
Microhemorrhage	19 (38.78)	1 (5.00)	18 (62.07)	< 0.001
Combined ABC score				
None	15 (30.61)	15 (75.00)	0 (0.00)	< 0.001
Low	19 (38.78)	4 (20.00)	15 (51.72)
Intermediate	6 (12.24)	0 (0.00)	6 (20.69)
High	9 (18.37)	1 (5.00)	8 (27.59)
A score				
Mean [SD]	1.35 [1.18]	0.35 [0.75]	2.07 [0.92]	< 0.001
0	15 (30.61)	15 (75.00)	0 (0.00)	< 0.001
1	15 (30.61)	4 (20.00)	11 (37.93)
2	6 (12.24)	0 (0.00)	6 (20.69)
3	13 (26.53)	1 (5.00)	12 (41.38)
B score				
Mean [SD]	1.67 [0.97]	1.05 [0.89]	2.10 [0.77]	< 0.001
0	6 (12.24)	6 (30.00)	0 (0.00)	< 0.001
1	15 (30.61)	8 (40.00)	7 (24.14)
2	17 (34.69)	5 (25.00)	12 (41.38)
3	11 (22.45)	1 (5.00)	10 (34.48)
C score				
Mean [SD]	0.78 [1.18]	0.10 [0.45]	1.24 [1.30]	< 0.001
0	32 (65.31)	19 (95.00)	13 (44.83)	< 0.001
1	4 (8.16)	0 (0.00)	4 (13.79)
2	5 (10.20)	1 (5.00)	4 (13.79)
3	8 (16.33)	0 (0.00)	8 (27.59)
Global Ecog score				
≤ 1	33 (67.35)	18 (90.00)	15 (51.72)	0.008
1-2	4 (8.16)	0 (0.00)	4 (13.79)
>2	12 (24.49)	2 (10.00)	10 (34.48)

*Number of participants missing data: *APOE* allele = 2 (4.08%).

### 3.2 The demographic data and pathological characteristics of CAA samples

There were 29 samples (59.18%) with CAA and 20 samples (40.82%) without CAA. Samples with CAA (81.41 ± 9.01) were significantly elder than those without CAA (66.30 ± 17.93, *p* < 0.001), whereas no significant differences in gender distribution and PMD were observed between these two groups. Regarding genetic factors, *APOE ϵ4* allele carrier rate was higher in the CAA group (*p* = 0.015), while *APOE ϵ2* allele frequency was comparable in the two groups (**Table 1**).

In neuropathological comorbidities, compared with no CAA group, the CAA group exhibited higher prevalence of AD (48.28%), LATE (82.76%), arteriolosclerosis (93.10%), microinfarction (44.83%) and microhemorrhage (62.07%) (*p* < 0.05). No significant differences were found in ARTAG, LBD, FTLD, infarction and large hemorrhage. The combination of ABC score and individual A, B, and C scores were increased in CAA group (*p* < 0.001), while without CAA group predominantly presented the lowest ABC grade (**Table 1**).

Stratified by CAA status, the without CAA group demonstrated a lower cognitive burden, with 90.00% of subjects presenting an Ecog score ≤ 1 and only 10.00% having scores > 2. In contrast, the CAA group displayed significantly worse cognitive performance, showing a lower proportion of individuals with Ecog ≤ 1 (51.72%) and a markedly higher proportion of cases with scores of 1-2 (13.79%) and > 2 (34.48%). The between-group difference in Ecog score distribution was statistically significant (*p* = 0.008).

**Figure 1:**
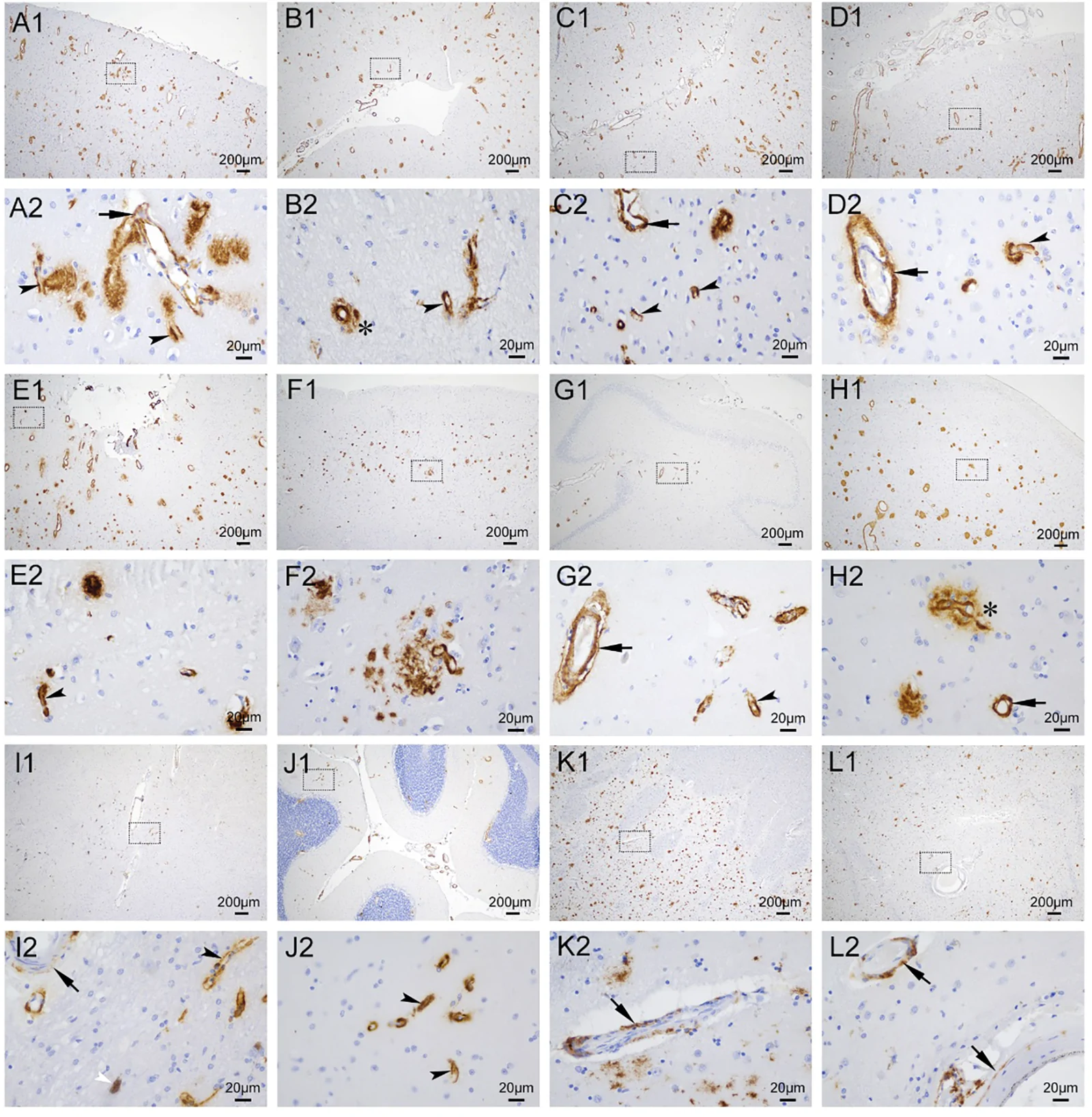
Examples of pathological CAA cases. Histochemical staining morphology of CAA vascular pathology from frontal cortex (A1, A2), temporal cortex (B1, B2), parital cortex (C1,C2), occipital cortex (D1, D2), insula cortex (E1, E2), entorhinal cortex (F1, F2), hippocampus (G1, G2), amygdala (H1, H2), substaintia nigra (I1, I2), cerebellum (J1, J2), caudata (K1, K2) and putamen (L1, L2). The area within the dashed box area the selected enlarged sections. Black arrows show the parenchymal vessels of neocortical areas, black arrowheads show the capillaries. Asterisk show the dyshoric change in CAA. White arrowhead in I2 shows the neuron in substaintia nigra. Bar = 200 μm in A1-L1, bar = 20 μm in A2-L2.

Immunohistochemical staining showed that CAA were seen in leptomeningeal and cortical arteries, and few to frequently in veins and capillaries as well as in subcortical vessels. In arteries and veins, Aβ depositions were attached to the outer basement membrane or occur between the smooth muscle cells of the lamina media. Capillary Aβ depositions were attached to the capillary basement membrane and frequently extend into the neuropil in a plaque-like manner, named “dyshoric angiopathy” (**Figure 1**). Among the 29 cases with CAA, the distribution of CAA stages was as follows: 19 (65.52%) cases in stage 1, 7 (24.14%) in stage 2, and 3 (10.34%) in stage 3. For CAA pathological subtype, 10 cases (34.48%) were associated with capillary as type 1, whereas 19 cases (65.52%) that Aβ deposited only in larger vessels were as CAA type 2. Most type 1 CAA cases presented with higher stage pathology, while type 2 CAA was limited to a relatively lower stage (*p* < 0.001) (**Table 2**).

**Table 2:** Association between pathological type and stage of CAA.

Subtype	CAA	Stage 1	Stage 2	Stage 3	*P* Value
	29 (100)	19 (38.78)	7 (14.29)	3 (6.12)	
Type 1	10 (20.41)	0	7	3	< 0.001
Type 2	19 (38.78)	19	0	0	

### 3.3 The correlation between CAA and ADNC

Correlation analyses were conducted to assess the associations of age, gender, and PMD with CAA related indices, ABC scores, and Ecog score. Data are presented as correlation coefficients *r* with corresponding *p* values. Age was significantly and positively associated with all outcome measures. Specifically, age was correlated with CAA type (*r*=0.483, *p*<0.001), CAA stage (*r*=0.455, *p*=0.001), combined ABC score (*r*=0.566, *p*<0.001), A score (*r*=0.546, *p*<0.001), B score (*r*=0.679, *p*<0.001), C score (r=0.495, *p*<0.001), and Ecog score (*r*=0.311, *p*=0.029). All correlations with age remained highly significant at the *p*<0.01 level except for cognitive function, which was significant at the *p*<0.05 level (two-tailed). Gender and PMD showed no significant correlation with any of the CAA indices, ABC scores, or cognitive function (**Table 3**).

**Table 3:** Spearman correlations between CAA type, CAA stage, ABC scores, global Ecog score and demographic variables.

Variables	CAA type	CAA stage	Combined ABC score	A score	B score	C score	Ecog score
Age	0.483**	0.455**	0.566**	0.546**	0.679**	0.495**	0.311*
	(<0.001)	(0.001)	(<0.001)	(<0.001)	(<0.001)	(<0.001)	(0.029)
Gender	0.005	-0.015	0.184	0.175	0.106	0.246	0.185
	(0.973)	(0.919)	(0.205)	(0.229)	(0.468)	(0.089)	(0.204)
PMD	-0.219	-0.209	-0.163	-0.019	-0.171	-0.157	-0.270
	(0.130)	(0.149)	(0.264)	(0.652)	(0.236)	(0.281)	(0.061)

The results are displayed as correlation coefficients (*p* value) in each cell. * Correlation was significant at the 0.05 level (2-tailed). ** Correlation was significant at the 0.01 level (2-tailed).

**Figure 2:**
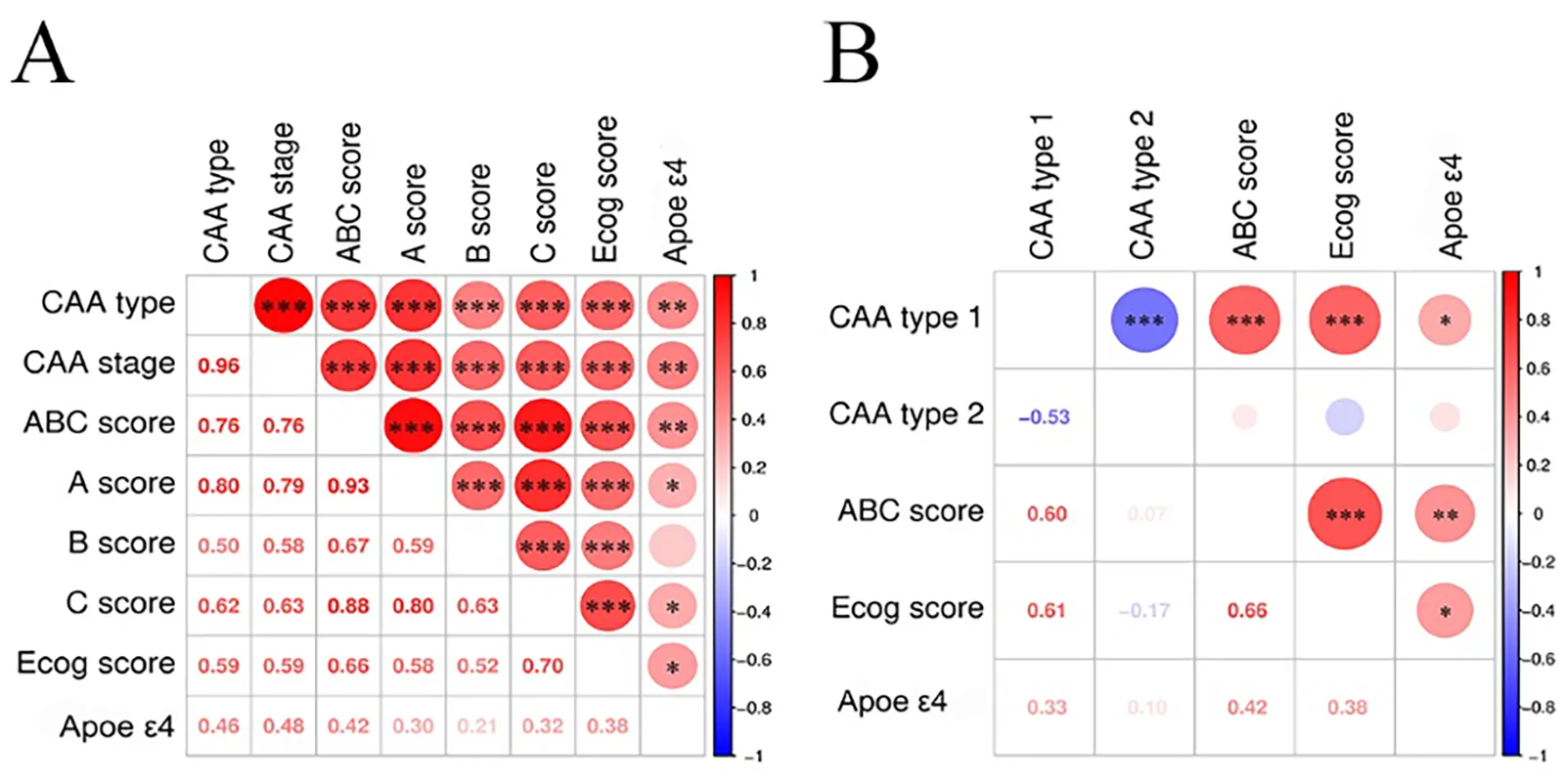
**(A)** Partial correlation heatmap of CAA pathological indicators, combined ABC score, Ecog score and *APOE ϵ4* genotype. Color gradient corresponds to correlation coefficient. **(B)** Partial correlations between CAA subtypes, ABC score, Ecog score and *APOE ϵ4.* *p<0.05, **p<0.01, ***p<0.001.

Partial correlation analyses were performed with adjustment for age. Notably, CAA type exhibited an extremely strong positive correlation with CAA stage (*r*=0.963, *p*<0.001). Both CAA type and stage were significantly and positively correlated with combined ABC score, subscale scores (A score, B score, C score), Ecog score (*p*<0.001). In terms of genetic correlation, the *APOE ϵ4* genotype was positively associated with CAA severity indicators, ABC scoring system indices and Ecog functional status (*p*<0.05). Nevertheless, no significant correlation was observed between *APOE ϵ4* status and B score (*r*=0.208, *p*=0.166) (**Figure 2A**).

Partial correlation analysis was performed to explore interrelationships among CAA subtypes distribution, ABC score, Ecog cognitive score and *APOE ϵ4* status. Significant moderate negative correlation was identified between CAA type 1 and CAA type 2 (*r*=−0.53, *p*<0.001), indicating an inverse distribution pattern of these two pathological CAA subtypes. CAA type 1 exhibited robust positive correlations with ABC score (*r*=0.605, *p*<0.001) and Ecog score (*r*=0.61, *p*<0.001), alongside a mild positive association with *APOE ϵ4* carrier status (*r*=0.33, *p*=0.027). In contrast, no statistically significant correlations were observed between CAA type 2 and ABC score (*r*=0.07, *p*=0.645), Ecog score (r=−0.17, *p*=0.263), or *APOE ϵ4* (*r*=0.10, *p*=0.494). ABC score was strongly positively correlated with Ecog cognitive impairment score (*r*=0.66, *p*<0.001). Higher ABC amyloid burden was significantly associated with *APOE ϵ4* positivity (*r*=0.42, *p*=0.003). Moreover, *APOE ϵ4* carriage was moderately positively associated with elevated Ecog score (*r*=0.38, *p*=0.010). Collectively, *APOE ϵ4* is linked to aggravated cognitive dysfunction possibly via promoting CAA type 1 pathology and increasing cerebral amyloid angiopathy burden (**Figure 2B**).

### 3.4 Differentially expressed genes of transcriptome analysis in CAA and CAA subtypes

**Figure 3:**
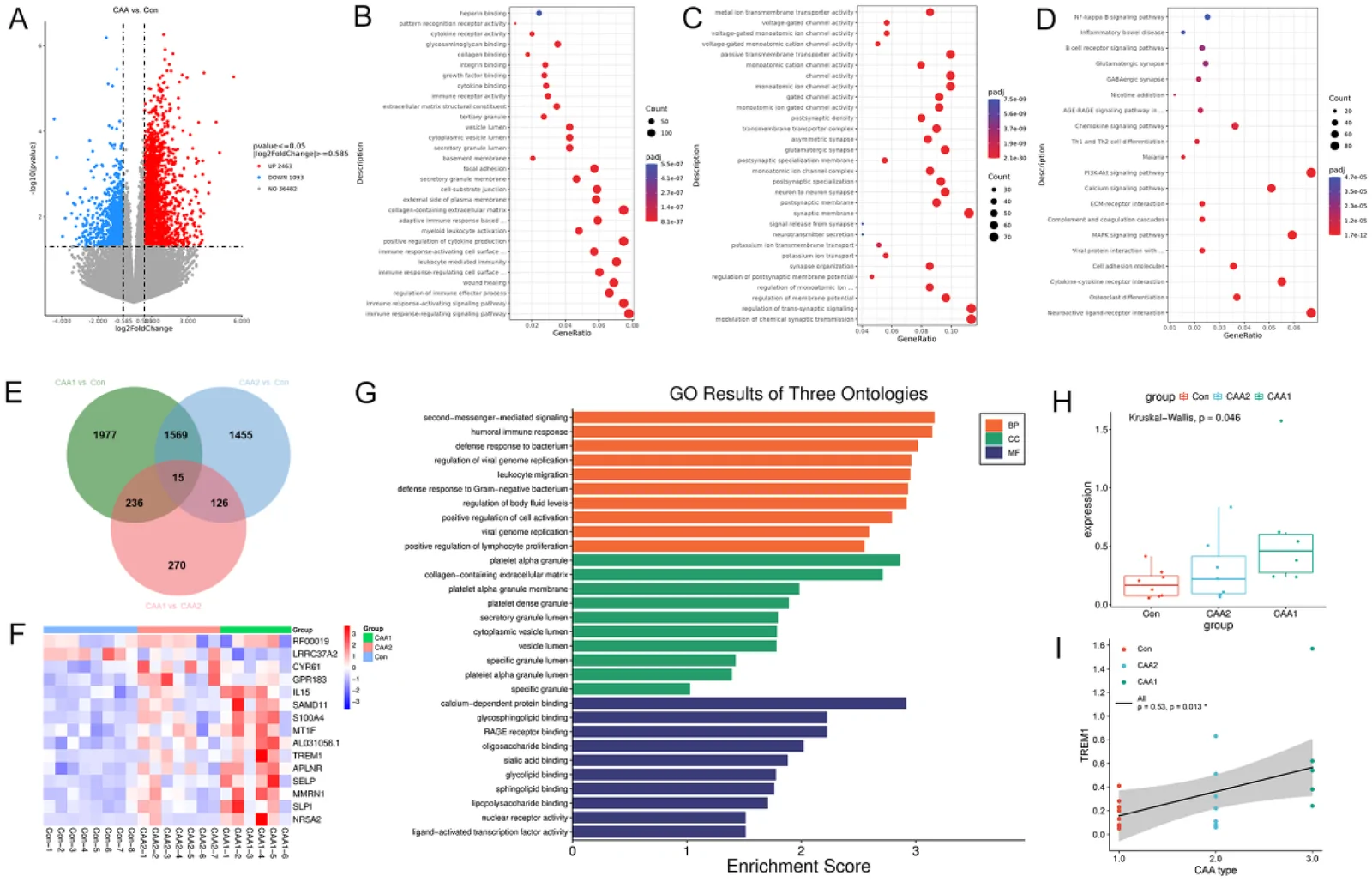
Differentially expressed genes of transcriptome analysis in CAA and CAA subtypes. **(A)** Volcano plot analysis of differentially expressed genes between the CAA group and the control group. **(B)** GO enrichment analysis based on up-regulated differential expressed genes. **(C)** GO enrichment analysis based on down-regulated differentially expressed genes. **(D)** KEGG enrichment analysis based on differentially expressed genes. **(E)** Venn diagram of differentially expressed genes among CAA1, CAA2 and control groups. **(F)** Heat map of 15 differentially expressed genes among CAA1, CAA2 and control groups. **(G)** GO enrichment analysis based on 15 differentially expressed genes. **(H)** Box-and-scatter plot of gene expression levels across the control, CAA2 and CAA1 groups. Kruskal-Wallis test revealed significant intergroup differences (p = 0.046). **(I)** Correlation between CAA subtypes and TREM1 expression levels. Spearman’s rank correlation analysis revealed a significant positive association (ρ=0.53, p=0.013). The black line represents the fitted trend line, and the grey shaded area indicates the 95% confidence interval.

Transcriptome data can be found in **Supplementary File 2**. There were 3556 differential expressed genes in the CAA dataset that met |logFC| > 0.585 and *p* value < 0.05 threshold. Under this threshold, 2463 genes were up-regulated and 1093 genes were down-regulated, showing in the volcano diagram (**Figure 3A**). For up-regulated genes (**Figure 3B**; **Supplementary File 3**), functional enrichment analysis of differential expressed genes was performed to dissect the underlying biological signatures. At the molecular function level, differential expressed genes were markedly enriched in extracellular matrix binding and immune receptor activities, including glycosaminoglycan, collagen, integrin, growth factor and cytokine binding, as well as pattern recognition, cytokine and immune receptor activities, with heparin binding being the sole term with relatively attenuated statistical significance. Cellular component enrichments highlighted secretory granule compartments, basement membrane, focal adhesion, cell-substrate junctions and the outer plasma membrane alongside collagen-containing extracellular matrix, indicative of disrupted granulocyte cargo release, cell-matrix anchorage and membrane immune complex assembly. Biological process terms exhibited the highest GeneRatio and extreme statistical significance, predominantly encompassing myeloid leukocyte activation, leukocyte-mediated immunity, adaptive immune response, positive regulation of cytokine production and wound healing; multiple immune response-modulating cell-surface signaling pathways harbored the largest gene sets and strongest enrichment signals. Cumulatively, these GO signatures demonstrate that differential expressed genes converge on extracellular matrix remodeling, granulocyte secretion, myeloid immune cell activation and immune signal transduction, establishing extracellular matrix homeostasis dysfunction and aberrant innate/adaptive immune cascades as core molecular hallmarks of the investigated pathological phenotype. For down-regulated genes (**Figure 3C**; **Supplementary File 4**), these GO signatures reveal that transcriptional activates cation transmembrane transport, excitatory synapse structural remodeling and chemical synaptic transmission cascades, identifying dysregulated ion channel homeostasis and augmented glutamatergic synaptic signaling as core neuronal molecular hallmarks of the pathological phenotype under investigation. KEGG-enriched pathways were categorized into three functional modules. The immune-inflammatory module dominated the statistical significance, including NF-κB, MAPK, PI3K-Akt, chemokine, cytokine-cytokine receptor interaction, complement and coagulation cascades. The neuronal synaptic module covered glutamatergic synapse, GABAergic synapse, calcium signaling pathway and neuroactive ligand-receptor interaction. The extracellular matrix and cell adhesion module contained ECM-receptor interaction and cell adhesion molecule pathways. Additionally, adaptive immune differentiation pathways (Th1/Th2 cell differentiation, B cell receptor signaling) and AGE-RAGE signaling were significantly enriched. Pathways with the largest gene counts and highest GeneRatio included neuroactive ligand-receptor interaction, PI3K-Akt, and MAPK signaling pathways, indicating these three cascades represent the most prominent transcriptional perturbations in differential expressed genes (**Figure 3D**; **Supplementary File 5**).

There were 15 different expressed genes among control, CAA2 and CAA1 groups (**Figures 3E, F**). GO enrichment analysis (**Figure 3G**; **Supplementary File 6**) revealed that differentially expressed genes were significantly enriched in terms related to immune response, leukocyte migration and collagen-containing extracellular matrix. Among them, *IL15, TREM1, GPR183, S100A4, MT1F*, and *NR5A2* were mainly associated with immune response by regulating inflammatory activation, immune cell proliferation, and pro-inflammatory signaling. *SELP, GPR183*, and *APLNR* were primarily involved in leukocyte migration through mediating leukocyte adhesion, chemotaxis, and transmigration. *CYR61, S100A4*, and *MMRN1* were closely linked to collagen-containing extracellular matrix by modulating extracellular matrix organization, cell adhesion, and matrix remodeling. We further validated the expression trend of *TREM1*, a key immune gene in immune-inflammatory responses. Kruskal-Wallis test confirmed significantly elevated *TREM1* expression in CAA subgroups compared with controls (**Figure 3H**, *p*=0.046). Linear regression fitting demonstrated a progressive upregulation of *TREM1* in CAA subtypes (**Figure 3I**, ρ=0.53, *p*=0.013).

### 3.5 Immunohistochemical staining of different gene in CAA subtypes (TREM1)

**Figure 4:**
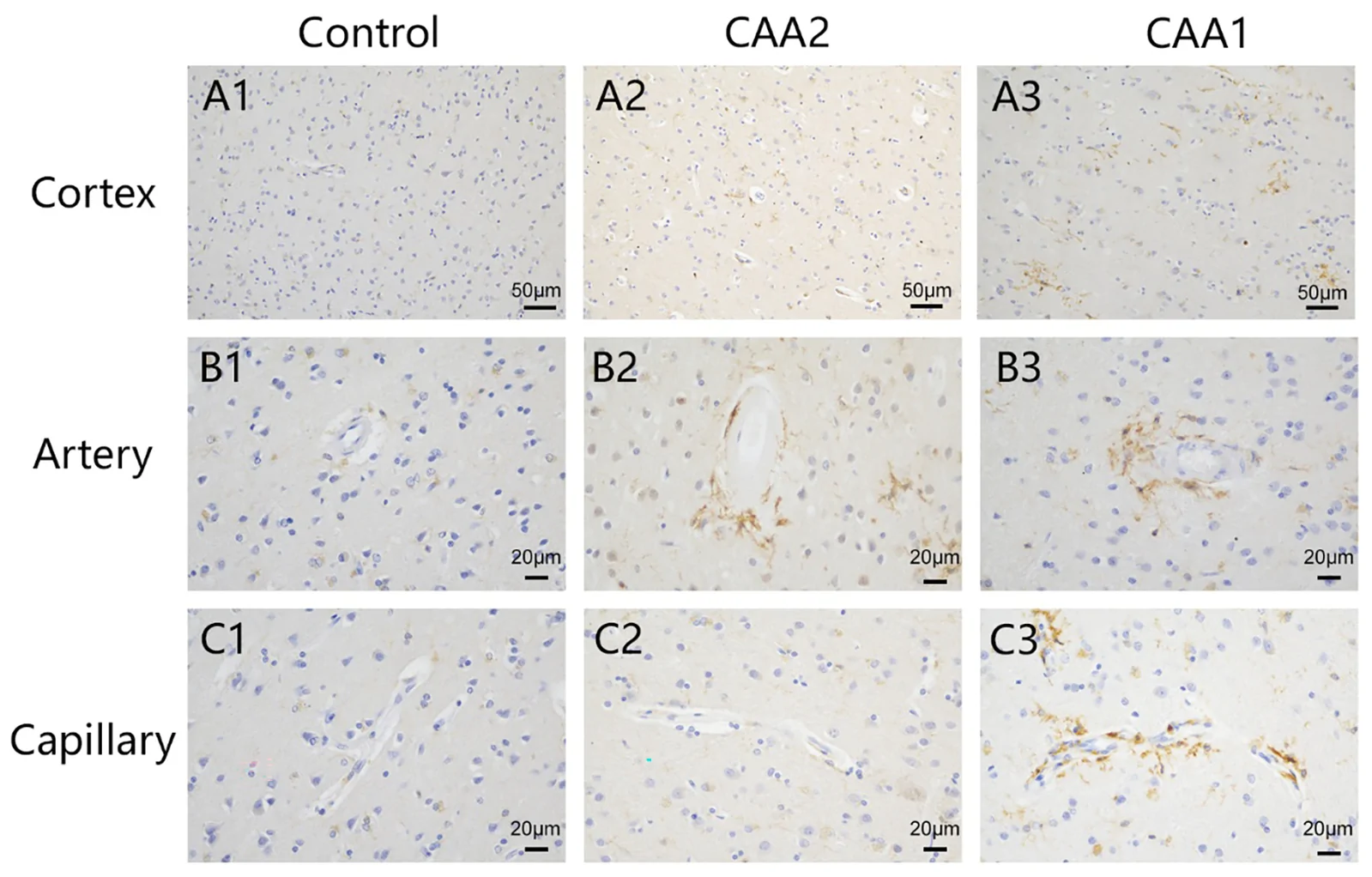
Immunohistochemical staining of TREM1 in CAA subtypes. Occipital cortex **(A1–A3)**, artery in the cortex **(B1–B3)**, capillary in the cortex **(C1–C3)**. Almost negative staining in control samples. Few positive microglia scattered in the cortex, or located around with arteries but without capillaries. Many plaques-like lesions scattered in the cortex with amyloid in the central core, also TREM1 positive microglia located around arteries and capillaries. Bar = 50 μm in A1-A31, bar = 20 μm in B1-C3.

Minimal TREM1 immunohistochemical staining was detected in brain sections from control samples; cortical blood vessels showed sharp, unlabeled vascular borders, and the perivascular brain parenchyma lacked any visible pathological lesions (**Figure 4A1, B1, C1**). In CAA2 group, a few weakly positive cells scattered within the parenchyma. Some positive cells located around the small arteries, but the pericapillary regions were negative. In contrast, robust upregulation and distinct pathological aggregation of TREM1 immunoreactivity were detected in CAA1 brain specimens. Within typical plaque lesions, TREM1 immunoreactivity encircled the central core, recapitulating the spatial distribution signature of Aβ plaques in cortices (**Figure 4A3**). Heavy brown TREM1 immunoreactive cells surrounded cortical microvessels and extended into the surrounding neural parenchyma (**Figure 4B3, C3**). TREM1-immunopositive cells within these lesions displayed hypertrophic somata and thick, truncated cellular processes.

## 4 Discussion

Existing epidemiological investigations of CAA have predominantly centered on Western cohorts, whereas systematic population-based autopsy data delineating the pathological and epidemiological characteristics of CAA in Chinese populations remain underrepresented. In the present autopsy cohort, the overall CAA prevalence was 59.18%, but the previously documented prevalence range of 16%-70% in the elderly population (10, 11). Such interethnic disparities are plausibly attributed to inherent differences in genetic predisposition, habitual lifestyle patterns, and exogenous environmental risk exposures across distinct populations. In line with canonical findings, individuals with CAA exhibited significantly advanced age compared with CAA-negative subjects, corroborating aging as the primary non-modifiable risk factor governing CAA pathogenesis. Meanwhile, no sex-dependent disparity in CAA prevalence was detected in our cohort, which is consistent with prior population-based observations (32). Patients with CAA harbored a markedly elevated burden of cerebral microhemorrhages, while the incidence of catastrophic macroscopic hemorrhages was statistically comparable between CAA-positive and CAA-negative groups. Although the lack of intergroup difference in macrohemorrhagic events may be partially confounded by limited sample size, this discrepant lesion distribution reveals a pivotal pathophysiological paradigm: cerebral microhemorrhages serve as highly sensitive early and subclinical surrogate markers of vascular amyloid accumulation, emerging long before irreversible structural vascular destruction and life-threatening macroscopic hemorrhage occur. As reported in previous studies, *APOE ϵ4* confers risk for sporadic CAA onset, whereas *APOE ϵ2* primarily increases the risk of CAA-associated hemorrhage rather than the overall prevalence of CAA (12). Our data showed elevated *APOE ϵ4* allele frequency in CAA patients, which is consistent with existing evidence. No significant difference in *APOE ϵ2* allele frequency was detected between groups, which may be explained by limited sample size and population-specific genetic background of our Chinese autopsy cohort. Furthermore, studies investigating CAA based on Chinese brain bank samples are still limited. A published study from another Chinese brain bank reported similar *APOE ϵ2* allele frequency and also failed to identify a significant association between the *APOE ϵ2* allele and CAA (32). We believe this is a promising scientific issue worthy of in-depth exploration in follow-up research. Larger cohorts should be established in future studies to validate findings related to the stratification, subtyping and grading of CAA.

Discussion correlation analysis demonstrated an extremely strong positive association between CAA type and stage, indicating high consistency between the two pathological grading systems. Both CAA type and stage were significantly and positively correlated with combined ABC score, subscale scores (A score, B score, C score), Ecog score and *APOE ϵ4* allele, indicating that aggravated CAA burden was closely linked to elevated clinical grading scores and poorer physical performance status. Notably, no significant correlation was detected between *APOE ϵ4* allele and subscore B, suggesting phenotypic heterogeneity in the genetic effect of *APOE ϵ4* allele across distinct CAA pathological dimensions. A major strength of our analysis lies in the strict application of Thal’s criteria to stratify CAA into type 1 (capillary involvement) and type 2 (restricted to leptomeningeal and cortical larger vessels) (23). We showed a profound topological and evolutionary divergence between these two phenotypes. Type 2 CAA cases were predominantly confined to early stages (stage 1), whereas type 1 CAA showed a dramatic propensity to progress to advanced stages (stages 2 and 3), involving allocortical, cerebellar, and subcortical structures. This stage-specific distribution pattern implies that capillary Aβ deposition serves as a pathological accelerator, likely driving a widespread collapse of the cerebrovascular network. The partial correlation analysis (adjusted for age) revealed that type 1 CAA carries a far stronger association with global ADNC burden (ABC scores) and poorer cognitive performance than type 2. This indicates that capillary dysfunction disrupts the blood-brain barrier and glymphatic clearance more catastrophically than large-vessel amyloidosis alone, fueling a vicious cycle of parenchymal Aβ retention and tau hyperphosphorylation (33).

In the CAA group, different expressed genes enriched in immune and extracellular matrix signatures demonstrated robust transcriptional activation of myeloid leukocyte function, granulocyte degranulation, cytokine secretion, and cell-matrix adhesion machinery. Upregulated genes indicated excessive innate immune infiltration and pro-inflammatory mediator release, which is a well-established upstream trigger for neuronal excitotoxic injury (36). Accumulated extracellular matrix components and elevated adhesive signaling may further amplify immune cell recruitment to the neural microenvironment, forming a self-perpetuating pro-inflammatory loop that disturbs parenchymal tissue homeostasis (37). Notably, the extremely low adjusted *p*-values across all immune-related biological process terms underscored that immune activation represents a dominant transcriptional perturbation in this pathological state. Complementarily, synaptic and ion channel-related GO terms highlighted severe disruptions to neuronal electrophysiology and excitatory synaptic architecture, which predisposes neurons to aberrant membrane depolarization and hyperexcitability. Concomitant enrichment of glutamatergic synapse, postsynaptic specialization, and chemical synaptic transmission modulation pathways further revealed structural remodeling of excitatory synapses and potentiated trans-synaptic signaling. Only two minor synaptic secretion terms exhibited attenuated statistical significance, suggesting the core synaptic pathology lies in postsynaptic ion channel dysfunction rather than presynaptic neurotransmitter release. Sustained myeloid immune activation and excess pro-inflammatory cytokine production can transcriptionally upregulate neuronal cation channel subunits and reshape postsynaptic glutamatergic scaffolds, exacerbating neuronal hyperexcitability and synaptic over-transmission. In turn, persistent excitotoxic synaptic signaling may further stimulate microglial/leukocyte activation and pro-inflammatory factor secretion, creating a vicious neuroinflammation-excitotoxicity cycle that propagates neural tissue damage. Mechanistically, NF-κB/MAPK cascades promote robust pro-inflammatory cytokine secretion (34), which activates PI3K-Akt and calcium signaling to potentiate excitatory synaptic transmission; in turn, persistent synaptic hyperexcitability stimulates complement and chemokine release to amplify neuroinflammation.

The Venn diagram illustrated substantial overlap of differentially expressed genes between CAA2 and CAA1 relative to control tissues, reflecting conserved molecular cascades triggered by initial vascular Aβ deposition. Notably, CAA1 harbored a vastly expanded unique differentially expressed genes pool compared with mild CAA2, demonstrating that CAA subtypes engages a broad, disease-subtype-specific transcriptional program that is largely absent in vascular amyloidosis. The curated immune and vasculopathy gene heatmap visually stratifies the dichotomous transcriptional states across disease subtypes. A core signature of 15 differentially expressed genes implies that they may exert pivotal biological effects by regulating immune homeostasis, leukocyte chemotaxis and extracellular matrix remodeling. This stepwise transcriptional amplification cannot be attributed to passive Aβ accumulation alone, instead, it indicates a feedforward inflammatory circuit that self-potentiates microglial reactivity. Vascular amyloid deposition triggers a restrained inflammatory response in CAA2. CAA1, however, activates a broad subtype-specific transcriptional program. This program sustains myeloid hyperactivation, endothelial injury and ECM remodeling, which impairs neurovascular function over time. These molecular differences explain uneven tissue vulnerability across CAA subtypes and uncover candidate therapeutic targets for severe CAA.

TREM1 is a myeloid-specific pro-inflammatory receptor that amplifies microglial reactivity via DAP12-NF-κB signaling, driving persistent tissue-damaging neuroinflammation upon amyloid stimulation (35). Severe CAA1 exhibited synchronous, marked overexpression of TREM1 paired with tissue-degrading mediators (MMP family) and inflammasome components, illustrating a phenotypic switch from surveillant microglia to a neurotoxic, tissue-destructive myeloid population. Meanwhile, robust TREM1 aggregation surrounded both cortical microvessels and parenchymal Aβ plaques, this dual spatial distribution indicates TREM1-driven myeloid inflammation is activated by both vascular and parenchymal amyloid pathology in advanced CAA. Our results unite transcriptomic and histopathological evidence to demonstrate that TREM1-mediated microglial inflammation escalates synchronously with CAA subtypes. Unlike TREM2, which mediates homeostatic Aβ clearance (38), TREM1 drives maladaptive chronic neuroinflammation that exacerbates tissue injury in high-grade CAA, rendering TREM1 a candidate histologic severity marker and potential therapeutic target for severe cerebral amyloid angiopathy.

Nevertheless, this study possesses several inherent limitations. First, all analyses are performed based on transcriptomic data, lacking validations at the protein and epigenetic levels. Second, the sample size of the enrolled cohorts is relatively small, which may weaken statistical power and limit the generalizability of our findings. Third, most conclusions are derived purely from bioinformatics mining, lack of sufficient supporting *in vitro* and *in vivo* experimental evidence. Further functional verification, such as gene knockdown to explore the effects of core genes on CAA-related cellular phenotypes, is required to confirm our computational findings. Fourth, although strict quality control and standardized normalization were conducted during data integration, potential batch effects across multiple datasets cannot be completely excluded.

In conclusion, the combined application of pathological grading and transcriptomic data analysis can achieve refined risk stratification for CAA. These prospective validations will lay a solid theoretical foundation for the development of novel risk diagnostic biomarkers and targeted therapeutic agents for CAA.

## Data Availability

The original contributions presented in the study are included in the article/**Supplementary Material**. Further inquiries can be directed to the corresponding authors.

## References

[1] Biffi A Greenberg SM . Cerebral amyloid angiopathy: a systematic review. J Clin Neurol. (2011) 7:1–9. doi: 10.3988/jcn.2011.7.1.1 21519520 PMC3079153

[2] Grinberg LT Thal DR . Vascular pathology in the aged human brain. Acta Neuropathol. (2010) 119:277–90. doi: 10.1007/s00401-010-0652-7 20155424 PMC2831184

[3] Greenberg SM Bacskai BJ Hernandez-Guillamon M Pruzin J Sperling R van Veluw SJ . Cerebral amyloid angiopathy and Alzheimer’s disease - one peptide, two pathways. Nat Rev Neurol. (2020) 16:30–42. doi: 10.1017/cbo9780511691836.005 31827267 PMC7268202

[4] Scheltens P De Strooper B Kivipelto M Holstege H Chételat G Teunissen CE . Alzheimer’s disease. Lancet. (2021) 397:1577–90. doi: 10.1016/S0140-6736(20)32205-4 PMC8354300 33667416

[5] Genisman RV Oksova EE . Morphological diagnosis of vascular and senile dementia (the importance of cerebral congophilic angiopathy). Neurosci Behav Physiol. (1989) 19(6):488–93. doi: 10.1007/BF01181864 2615963

[6] Joachim CL Morris JH Selkoe DJ . Clinically diagnosed Alzheimer’s disease: autopsy results in 150 cases. Ann Neurol. (1988) 24:50–6. doi: 10.1002/ana.410240110 3415200

[7] Parker JC Jr Philpot J . Postmortem evaluation of Alzheimer’s disease. South Med J. (1985) 78:1411–3. doi: 10.1097/00007611-198512000-00003 4071164

[8] Ritter MA Droste DW Hegedüs K Szepesi R Nabavi DG Csiba L . Role of cerebral amyloid angiopathy in intracerebral hemorrhage in hypertensive patients. Neurology. (2005) 64:1233–7. doi: 10.1212/01.wnl.0000156522.93403.c3 15824353

[9] De Reuck J . The impact of cerebral amyloid angiopathy in various neurodegenerative dementia syndromes: a neuropathological study. Neurol Res Int. (2019) 2019:7247325. doi: 10.1155/2019/7247325 30792924 PMC6354160

[10] Tanskanen M Mäkelä M Myllykangas L Notkola IL Polvikoski T Sulkava R . Prevalence and severity of cerebral amyloid angiopathy: a population-based study on very elderly Finns (Vantaa 85+). Neuropathol Appl Neurobiol. (2012) 38:329–36. doi: 10.1111/j.1365-2990.2011.01219.x 21916927

[11] Robinson JL Corrada MM Kovacs GG Dominique M Caswell C Xie SX . Non-Alzheimer’s contributions to dementia and cognitive resilience in The 90+ Study. Acta Neuropathol. (2018) 136:377–88. doi: 10.1007/s00401-018-1872-5 29916037 PMC6534149

[12] O’Donnell HC Rosand J Knudsen KA Furie KL Segal AZ Chiu RI . Apolipoprotein E genotype and the risk of recurrent lobar intracerebral hemorrhage. N Engl J Med. (2000) 342(4):240–45. doi: 10.1056/NEJM200001273420403 10648765

[13] Ullah R Lee EJ . Advances in amyloid-β clearance in the brain and periphery: implications for neurodegenerative diseases. Exp Neurobiol. (2023) 32(4):216–46. doi: 10.5607/en23014 PMC10569141 37749925

[14] Morel F . Petite contribution a l‘etude d’une angiopathie apparemment dyshorique et topistique. Rev Mens Psychiatr Neurol. (1950) 120:352–7. doi: 10.1159/000148423 14806299

[15] Chen Y He Y Han J Wei W Chen F . Blood-brain barrier dysfunction and Alzheimer’s disease: associations, pathogenic mechanisms, and therapeutic potential. Front Aging Neurosci. (2023) 15:1258640. doi: 10.3389/fnagi.2023.1258640 38020775 PMC10679748

[16] Parodi-Rullán RM Javadov S Fossati S . Dissecting the crosstalk between endothelial mitochondrial damage, vascular inflammation, and neurodegeneration in cerebral amyloid angiopathy and Alzheimer's disease. Cells. (2021) 10:2903. doi: 10.3390/cells10112903 34831125 PMC8616424

[17] Kolobova E Petrushanko I Mitkevich V Makarov AA Grigorova IL . β-amyloids and immune responses associated with Alzheimer’s disease. Cells. (2024) 13:1624. doi: 10.3390/cells13191624 39404388 PMC11475064

[18] Henningfield CM Arreola MA Soni N Spangenberg EE Green KN . Microglia-specific ApoE knock-out does not alter Alzheimer’s disease plaque pathogenesis or gene expression. Glia. (2022) 70:287–302. doi: 10.1002/glia.24105 34643971 PMC9308171

[19] van Lengerich B Zhan L Xia D Chan D Joy D Park JI . A TREM2-activating antibody with a blood-brain barrier transport vehicle enhances microglial metabolism in Alzheimer's disease models. Nat Neurosci. (2023) 26:416–29. doi: 10.1038/s41593-022-01240-0 36635496 PMC9991924

[20] Ebrahimi R Nejad SS Tafti MF Karimi Z Sadr SR Hussein DR . Microglial activation as a hallmark of neuroinflammation in Alzheimer’s disease. Metab Brain Dis. (2025) 40:207. doi: 10.1007/s11011-025-01631-9 40381069

[21] Wang X Wu J Wang N Zhang D Chen Z Zhang H . Standardized operational protocol for the China human brain bank consortium. Hum Brain. (2022) 1:92–106. doi: 10.37819/hb.001.001.0209

[22] Qiu W Zhang H Bao A Zhu K Huang Y Yan X . Standardized operational protocol for human brain banking in China. Neurosci Bull. (2018) 35:270–6. doi: 10.1007/s12264-018-0306-7 30411305 PMC6426885

[23] Thal DR Griffin WST de Vos RAI Ghebremedhin E . Cerebral amyloid angiopathy and its relationship to Alzheimer’s disease. Acta Neuropathol. (2008) 115:599–609. doi: 10.1007/s00401-008-0366-2 18369648

[24] Montine TJ Phelps CH Beach TG Bigio EH Cairns NJ Dickson DW . National Institute on Aging-Alzheimer’s Association guidelines for the neuropathologic assessment of Alzheimer’s disease: a practical approach. Acta Neuropathol. (2012) 123:1–11. doi: 10.1007/s00401-011-0910-3 22101365 PMC3268003

[25] Kovacs GG Ferrer I Grinberg LT Alafuzoff I Attems J Budka H . Aging-related tau astrogliopathy (ARTAG): harmonized evaluation strategy. Acta Neuropathol. (2016) 131:87–102. doi: 10.1007/s00401-015-1509-x 26659578 PMC4879001

[26] Ferrer I Santpere G Leeuwen FW . Argyrophilic grain disease. Brain. (2008) 131. doi: 10.3389/conf.neuro.01.2009.16.123 18234698

[27] Nelson PT Dickson DW Trojanowski JQ Jack CR Boyle PA Arfanakis K . Limbic-predominant age-related TDP-43 encephalopathy (LATE): consensus working group report. Brain. (2019) 142:1503–27. doi: 10.1093/brain/awz099 31039256 PMC6536849

[28] Nelson PT Lee EB Cykowski MD Alafuzoff I Arfanakis K Attems J . LATE-NC staging in routine neuropathologic diagnosis: an update. Acta Neuropathol. (2023) 145:159–73. doi: 10.1007/s00401-022-02524-2 36512061 PMC9849315

[29] Koga S Dickson DW . Recent advances in neuropathology, biomarkers and therapeutic approach of multiple system atrophy. J Neurol Neurosurg Psychiatry. (2018) 89:175–84. doi: 10.1136/jnnp-2017-315813 28860330

[30] Farias ST Mungas D Reed BR Cahn-Weiner D Jagust W Baynes K . The measurement of everyday cognition (ECog): scale development and psychometric properties. Neuropsychology. (2008) 22:531–44. doi: 10.1037/0894-4105.22.4.531 18590364 PMC2877034

[31] Zhang W Zhang Q Yang Q Liu P Sun T Xu Y . Contribution of Alzheimer’s disease neuropathologic change to the cognitive dysfunction in human brains with Lewy body-related pathology. Neurobiol Aging. (2020) 91:56–65. doi: 10.1016/j.neurobiolaging.2020.02.022 32224069

[32] Yin XS Sun J Wang X Wu W Chen Z Zhang D . Prevalence of cerebral amyloid angiopathy and its correlation with Alzheimer’s disease and cognition in an autopsy-confirmed cohort from China. Alzheimer’s Dement (Amst). (2025) 17:e70100. doi: 10.1002/dad2.70100 40201593 PMC11973253

[33] Weller RO Subash M Preston SD Mazanti I Carare RO . Perivascular drainage of amyloid- Peptides from the brain and its failure in cerebral amyloid angiopathy and Alzheimer’s disease. Brain Pathol. (2008) 18:253–66. doi: 10.1016/s0002-9440(10)65616-7 18363936 PMC8095597

[34] Chen L Deng H Cui H Fang J Zuo Z Deng J . Inflammatory responses and inflammation-associated diseases in organs. Oncotarget. (2017) 9:7204–18. doi: 10.18632/oncotarget.23208 29467962 PMC5805548

[35] Trivedi N Bhosale JD Pant A Suryawanshi SP Tiwari P Abel PW . Triggering receptor expressed on myeloid cells-1 (TREM-1) in inflammation and disease: mechanisms, therapeutic potential, and future directions. Int J Mol Sci. (2025) 26:10386. doi: 10.3390/ijms262110386 41226426 PMC12608911

[36] Jiang J Yu Y Kinjo ER Du Y Nguyen HP Dingledine R . Suppressing pro-inflammatory prostaglandin signaling attenuates excitotoxicity-associated neuronal inflammation and injury. Neuropharmacology. (2019) 149:149–60. doi: 10.1016/j.neuropharm.2019.02.011 30763657 PMC6486887

[37] Wareham LK Calkins DJ . Making tracks: microglia and the extracellular matrix. Mol Neurodegener. (2025) 20:101. doi: 10.1186/s13024-025-00898-x 41024112 PMC12481997

[38] Ulland TK Song WM Huang SCC Ulrich JD Sergushichev A Beatty WL . TREM2 maintains microglial metabolic fitness in Alzheimer’s disease. Cell. (2017) 170:649–663.e13. doi: 10.1016/j.cell.2017.07.023 28802038 PMC5573224

